# Medication-Related Errors Among Nurses by Unit Adaptation Levels: Bayesian Network–Based Exploratory Study

**DOI:** 10.2196/87436

**Published:** 2026-07-07

**Authors:** Naotaka Sugimura, Katsuhiko Ogasawara

**Affiliations:** 1 Department of Nursing, School of Health Sciences, Tokyo University of Technology, Ota-ku, Tokyo, Japan; 2 Graduate School of Health Sciences, Hokkaido University, Sapporo, Hokkaido, Japan; 3 Faculty of Health Sciences, Hokkaido University, N12-W5, Kita-ku, Sapporo, Hokkaido, 060-0812, Japan, 81 11-706-3409; 4 Graduate School of Engineering, Muroran Institute of Technology, Muroran, Hokkaido, Japan

**Keywords:** adaptive performance, artificial intelligence, Bayesian network modeling, data-driven education, patient safety

## Abstract

**Background:**

Medical errors occur more frequently in health care than in other industries due to challenges in patient safety education for nurses and students. To address this, it is important to identify the factors and structures underlying clinical errors and apply these insights to educational programs. Medication-related errors are highly preventable with appropriate interventions, highlighting the importance of data-driven safety education. Previous research suggests that unit adaptation, rather than clinical experience alone, plays a critical role in error occurrence. Focusing on “adaptive performance,” an underexplored concept in nursing, can help identify new educational strategies and interventions.

**Objective:**

This hypothesis-generating study used Bayesian network modeling to examine how unit experience—and, secondarily, total nursing experience—relates to the structure of medication-related errors, with implications for data-driven patient safety education and nursing student instruction.

**Methods:**

This mixed methods study conducted a qualitative root cause analysis of medication error reports to identify causal factors. Bayesian network modeling, an artificial intelligence–based approach, was used to visualize error-generation flows and compare models based on years of experience within the current unit. Data were obtained from 2023 medication-related error reports submitted by nurses to the Japan Council for Quality Health Care.

**Results:**

Among 119 medication-related incidents, the largest proportion occurred in internal medicine departments (n=47 cases, 39.5%), followed by surgical departments (n=27 cases, 22.7%), the intensive care unit (n=10 cases, 8.4%), and the emergency room (n=7 cases, 5.9%). Most incidents occurred in patient rooms or wards (n=97 cases, 81.5%). Root cause analysis identified 10 types of medication-related events and 23 contributing factors. Cases were categorized into low-, moderate-, and high-adaptation groups based on years of experience in the current unit. In the low-adaptation group (n=33), 69.7% of nurses had ≥5 years of total nursing experience, indicating that the group did not consist solely of novice nurses. The average entropy of incident events across the Bayesian network models ranged from 0.37 to 0.78, suggesting moderate to relatively high uncertainty in the inferred error structures. The moderate- and high-adaptation models exhibited fewer complex error networks, with weaker chains of unsafe conditions or actions than the low-adaptation model. The extensive clinical experience did not always prevent errors; rather, it was often linked to lapses in verification behavior.

**Conclusions:**

Unit adaptation appears to play a critical role in shaping error pathways. Nurses with lower unit adaptation demonstrated more complex error structures, indicating higher vulnerability to incident chains. In contrast, total years of nursing experience did not uniformly reduce risk and may even relate to lapses in verification behaviors.

## Introduction

Data-driven education is an approach that continuously enhances the quality of instruction and learning by analyzing data generated throughout the educational process [1,2]. In recent years, data-driven education has been increasingly applied to evaluate instructional quality by assessing learning outcomes [3]. However, in nursing education, it is particularly important to utilize real-world data not only for analyzing learner performance but also for informing the design and improvement of educational systems. Patient safety education is a representative example as it remains an underdeveloped area of curriculum design worldwide, with evident challenges in the early stages of program development. Despite the World Health Organization (WHO)’s concern over the high frequency of medical errors in health care compared with other industries [4], patient safety education remains marginal in nursing curricula, often addressed only informally or as part of broader topics, and research on educational interventions is limited [5,6].

At the core of this issue lies the fundamental nature of health care professionals as practitioners whose work directly influences clinical practice, aligned with indispensable clinical competencies. In response, the American Association of Colleges of Nursing has proposed a framework for competency-based education [7]. Grounded in the principle of “beginning with the end in mind” [8,9], competency-based education is essential for defining learning outcomes and designing effective backward curriculum in patient safety education [10-12]. Nurses are positioned at the frontline of patient care and bear critical responsibility as the final safeguard against medical errors and adverse events. However, in the absence of a well-established and systematic educational framework, they often depend heavily on on-the-job training. This imbalance in educational approaches may ultimately undermine efforts to ensure patient safety.

Among medical errors, medication-related errors are the most preventable and represent an area in which educational interventions can yield substantial improvement [13,14]. A previous study employing Bayesian network modeling (BNM) identified multiple knowledge-, behavior-, and environment-related factors that contribute to medication errors among nurses [15]. These findings indicate that many of these factors can be modified through education, suggesting the potential for significant error reduction. In particular, unfamiliarity with unit-specific tasks and insufficient adaptation to work routines have emerged as key contributors, underscoring the importance of unit adaptation.

Adaptive performance—the ability to respond flexibly to unexpected events and environmental changes [16]—plays a critical role in error occurrence. Medication errors in nursing can be understood as outcomes of performance shaped by fatigue, workload, and environmental conditions [17-19]. Two attribute variables have commonly been used to represent a nurse’s experience: total years of nursing experience and years of unit experience. The former is well known from the Benner classification of nursing expertise [20], which applied the 5-stage model and has informed criteria in some clinical ladders [21]. By contrast, unit adaptation has received limited attention in nursing and medical research. Consequently, much of the patient safety literature has focused on novice nurses with limited experience [22-25]. However, understanding the developmental stages and individual differences of adaptive performance is essential for designing effective educational strategies for both nurses and nursing students [26]. Focusing on unit adaptation [16] challenges the view that “inexperience” is defined solely by fewer years of clinical practice and enables a more precise identification of targets for educational interventions.

Accordingly, this hypothesis-generating study used BNM to examine how unit experience—and, secondarily, total nursing experience—relates to the structure of medication-related errors, with implications for data-driven patient safety education and nursing student instruction.

## Methods

### Study Design

This study used a mixed methods approach consisting of 2 main analytical processes: qualitative factor extraction and relational analysis. First, episodes of medication-related errors involving nurses were qualitatively analyzed using root cause analysis (RCA) to identify the contributing factors [15]. Next, the data were categorized into 3 groups based on years of unit experience: low adaptation (>1 y), moderate adaptation (1 to >3 y), and high adaptation (3 years or more). For each group, BNM was conducted to evaluate the relationships between the factors and compare the structural differences in the flow of medication-related errors.

### Data Sources and Data Selection

This study used data from the Medical Error Database published by the Japan Council for Quality Health Care (JCQHC) [27]. The JCQHC undertakes various initiatives, including the collection of medical errors and near-miss cases, to maintain public trust in health care services and improve their quality. The database includes reports from a total of 1899 medical institutions across Japan. The JCQHC requires participating medical institutions to report incidents that fall into the following categories (1–3). (1) Cases in which inappropriate medical care or management was clearly identified and resulted in patient death, permanent physical or psychological impairment, or the need for unexpected or more extensive treatment than anticipated. (2) Cases in which it could not be conclusively determined whether inappropriate medical care or management occurred, but which were otherwise comparable to category (1). (3) Cases other than (1) and (2) that provide useful information for preventing and reducing the recurrence of incidents within health care institutions. The reporting form of the JCQHC requires detailed documentation, including the patient’s condition and age group, the working status of the staff involved in the error, the circumstances leading to the incident, and measures for future prevention. From this database, 119 cases were selected for analysis based on the following criteria (): (1) a nurse was involved, (2) the error was medication-related, involving adverse events or equivalent medical errors, and (3) the case included sufficient contextual information to allow for RCA.

### Competency and Total Clinical Experience

Although there is no universally accepted definition of nursing competency, it is commonly described as “a core ability required to fulfill nursing responsibilities” [28] or as “the knowledge, behaviors, judgment, skills, values, and attitudes necessary to provide safe, effective, and high-quality care” [29]. These definitions are grounded in experiential learning theory [20] and suggest that competency is a transferable ability developed through the integration of knowledge and skills. In many studies, total years of clinical experience and length of employment are frequently treated as variables that influence competency development [20,30-32]. In this study, informed by the Benner 5-stage model [20] and its application within clinical ladder systems in the world and Japan [21,33], total years of nursing experience were treated as categorical data (<5 y, 5–10 y, and ≥10 y).

### Adaptive Performance and Current Unit Experience

In contrast to competency, performance, particularly work performance, is defined as “behaviors or actions that are relevant to the goals of the organization” [34] and as “scalable actions, behaviors, and outcomes” [35]. Work performance is categorized into 4 domains: task, contextual, counterproductive, and adaptive [36]. Fatigue and burnout are closely associated with work performance among nurses [17], which is a critical element influencing patient safety and holds substantial importance in clinical practice.

Adaptive performance refers to “the extent to which an individual adapts to changes in a work system or work roles” [16]. Although this concept has received limited attention in medical and nursing research, it is theoretically associated with experiential learning [21]. In this study, years of current unit experience were treated as categorical data (low adaptation: <1 y; moderate adaptation: 1 to <3 y; high adaptation: ≥3 y) as a pragmatic proxy for the degree of adaptation to a specific clinical environment, informed by the application of clinical ladder systems in Japan and internationally [21,33]. These 3 groups were intended to correspond to levels of clinical practice and functional independence in the workplace and were operationally defined as follows:

- Low adaptation: the stage of learning all tasks with instructions and follow-ups
- Moderate adaptation: the stage of learning some tasks with instructions and follow-ups while performing other tasks independently
- High adaptation: the stage of performing most tasks independently

### Analysis

#### RCA and Factor Extraction

A total of 119 medical error reports were reviewed to classify events and extract contributing factors. Although the primary coding process was conducted by one researcher, the overall analytical framework was developed through discussions between 2 authors. To minimize subjectivity in factor extraction, a fact-based analytical strategy was adopted. Specifically, contributing factors were strictly derived from observable descriptions in the incident reports rather than from interpretative judgments. The following RCA-guiding questions were applied to ensure systematic extraction: “Why did this event occur?,” “What actions or conditions involving the nurse or patient contributed to the event?” “If the event could have been prevented, what actions should the nurse have taken?” In addition, predefined factor definitions were established in advance (Table 1), and contributing factors were extracted as objective facts from the free-text descriptions regarding the event sequence and implemented countermeasures based on consistent criteria. Although some reports included subjective statements—such as the reporter’s psychological state or fatigue—these were excluded from the analysis because such descriptions varied among reports and could not ensure objective evaluation. The extracted factors were categorized into 4 groups: attributive, system-related, situational, and knowledge/behavioral. Each factor was labeled within its respective categories. Event classification and factor extraction based on the RCA were reported in our previous study [15], and their validity was confirmed through consistency with prior research [37].

**Table 1.** Explanations of nodes.

Node	Explanation
Event	
Wrong dose	Administered the wrong dose of medication
Wrong patient	Administered medicine to wrong patient
Improper mixing	Performed improper mixing
Wrong or unnecessary administration	Administered a different medication to the one that should have been administered or administered a medication unnecessarily
Overlooking contraindication	Missed contraindicated medication
Accidental ingestion/ overdose	Patient accidentally ingested or overdosed
Incorrect storage	Stored medicine in an incorrect storage method
Wrong timing	Administered medicine at the wrong time
Default of medication discontinue	Failed to discontinue medicine
Wrong route	Administered medicine by the wrong route
Attributional factors	Factors related to the attributes of nurses who made errors
Year as registered nurse	Divided into 5 years as registered nurse
<5	Including novice, advanced beginner, and competent nurse
5‐9	Including proficient nurse
≥10	Including expert nurse
Year as current unit	Divided into 3 stages of learning the tasks of a typical Japanese hospital unit
<1	The stage of learning all tasks with instructions and follow-ups
1‐3	The stage of learning some tasks with instructions and follow-ups, but performing some tasks independently
>3	The stage of performing most tasks independently
System factor	Factors related to the organization’s systems, such as electronic medical records and educational systems
Failure to detect by the electronic system	Not equipped with the ability to detect on electronic medical record system
Conditional factors	Factors related to conditions surrounding nurses. For example, the time of day, the type of work, and other conditions when errors had occurred
Weekday/holiday	Occurred during weekday or holiday
Work time	Work time was divided into 3 parts according to the patient’s life and the nurse’s work behavior
9 AM-4 PM	
4 PM-9 PM	
9 PM-9 AM	
Day shift/night shift	Occurred during day shift or night shift
Patients’ age (y)	Age of patients associated with errors
<20	
20‐59	
≥60	
Time pressure	Time pressure was present due to sudden emergencies or multiple tasks of the patient
Sudden change in patients’ schedule	There was a sudden change in schedule for examination or treatment
Failure of doctor’s orders	There was a failure of doctor’s orders.
Patients who do not follow instructions	Patient did not follow the instructions of health care professionals
Knowledge and behavioral factors	Factors related to nurses’ knowledge state and behavior.
Failure to confirm the 5 rights	Had not checked the 5 rights
Assumptions and forgetfulness	Assumed or forgot instructions and orders
Invalid double check	Nurses double-checking each other was invalid
Improper use of instruments and equipment	The use of instruments and equipment induced errors
Insufficient knowledge of medications	Did not have sufficient knowledge
Unfamiliarity with operations of medications	Did not know how to properly handle medications due to unfamiliarity
Failure of information communication	Discrepancies in communication when conveying information
Improper handling of medications	Handled medicine in the wrong way
Failure of monitoring	Monitoring was a possibly avoidable error
Task interruption	Task interrupted when error occurs
Non-reconfirming of inappropriate doctor’s orders	Did not check again even though nurse felt the doctor’s instructions were inappropriate
Lack of explanation to patients	Not enough explanation about medication to the patient
Miscalculation	Miscalculated appropriate medication dosage

#### Bayesian Network Modeling

BNM is an artificial intelligence (AI)–based probabilistic approach that represents qualitative dependencies among multiple variables through a graphical structure and expresses quantitative relationships using conditional probabilities [15]. In this study, each event and contributing factor was represented as a “node,” and causal or temporal relationships were indicated by “edges” connecting these nodes. The analysis was performed using BAYOLINK version 7.0.1 (NTT DATA Mathematical Systems), employing a greedy algorithm with an Akaike information criterion threshold of 0.02 [38]. We used this algorithm and criterion to avoid overlooking even weak associations between incidents and contributing factors, as well as relationships among the factors themselves, because this study was positioned as a hypothesis-generating investigation. While previous patient safety research has primarily relied on qualitative analyses based on individual incident case reviews or simple descriptive surveys, this study aims to explore hypotheses regarding common structural patterns underlying specific incident occurrences and the characteristics of contributing factors derived directly from the data.

Model construction combined algorithmic computation with expert judgment [15]. A major advantage of this method is its ability to infer potential causal relationships from relatively small datasets based on information criteria. During the process of model development, whitelist and blacklist constraints were applied to refine the network structure. No whitelist was specified, and the initial blacklist included attributive and system-related factors, which were conceptually treated as root-level nodes without parent relationships. This classification was informed by human factors–based frameworks [37,39]. These constraints were iteratively updated to remove irrelevant links and maintain logical consistency in the causal and temporal relationships. Final models were constructed for each adaptation group.

Unit experience was classified into 3 adaptation levels based on the general clinical ladder for nurses in Japan: low adaptation (<1 y), moderate adaptation (≥1 and <3 y), and high adaptation (≥3 y) [15]. The low-adaptation group was defined as the stage in which nurses performed all tasks under instruction and supervision. The moderate-adaptation group was defined as the stage in which nurses performed some tasks independently, while still requiring instruction and follow-up for others. The high-adaptation group was defined as the stage in which nurses performed most tasks independently. Consistent with the secondary objective of this study, when the node “Year as registered nurse” (categorical variable: <5 y, 5–9 y, and ≥10 y) was included in a model, the category associated with medical error occurrence was identified. The structural differences between the 3 models were evaluated based on the number of layers and the number and characteristics of factors not directly associated with adverse events or equivalent medical errors.

The average entropy was calculated as an indicator of uncertainty in BNM. When the child node is a binary variable, the average entropy ranges from 0 to 1.0, where lower values indicate lower uncertainty. For example, when the cross-tabulation between a parent node A (binary) and a child node B (binary) is completely uniform, the uncertainty reaches its maximum and the value becomes 1.0. A smaller value of the average entropy indicates that the state of the child node can be more easily determined, given the condition of the parent node. Although there is no universally established threshold for interpreting the magnitude of average entropy, in this study, the average entropy was calculated only for binary nodes, and therefore, the values were restricted to the range of 0 to 1.0. Accordingly, the model was evaluated comparatively within this range.

### Ethical Considerations

This study used data from the anonymized and publicly available database of the JCQHC [26]. Therefore, it did not violate any privacy policies and did not fall under the scope of an ethics review.

## Results

### Descriptive Statistics

**Figure 1.**
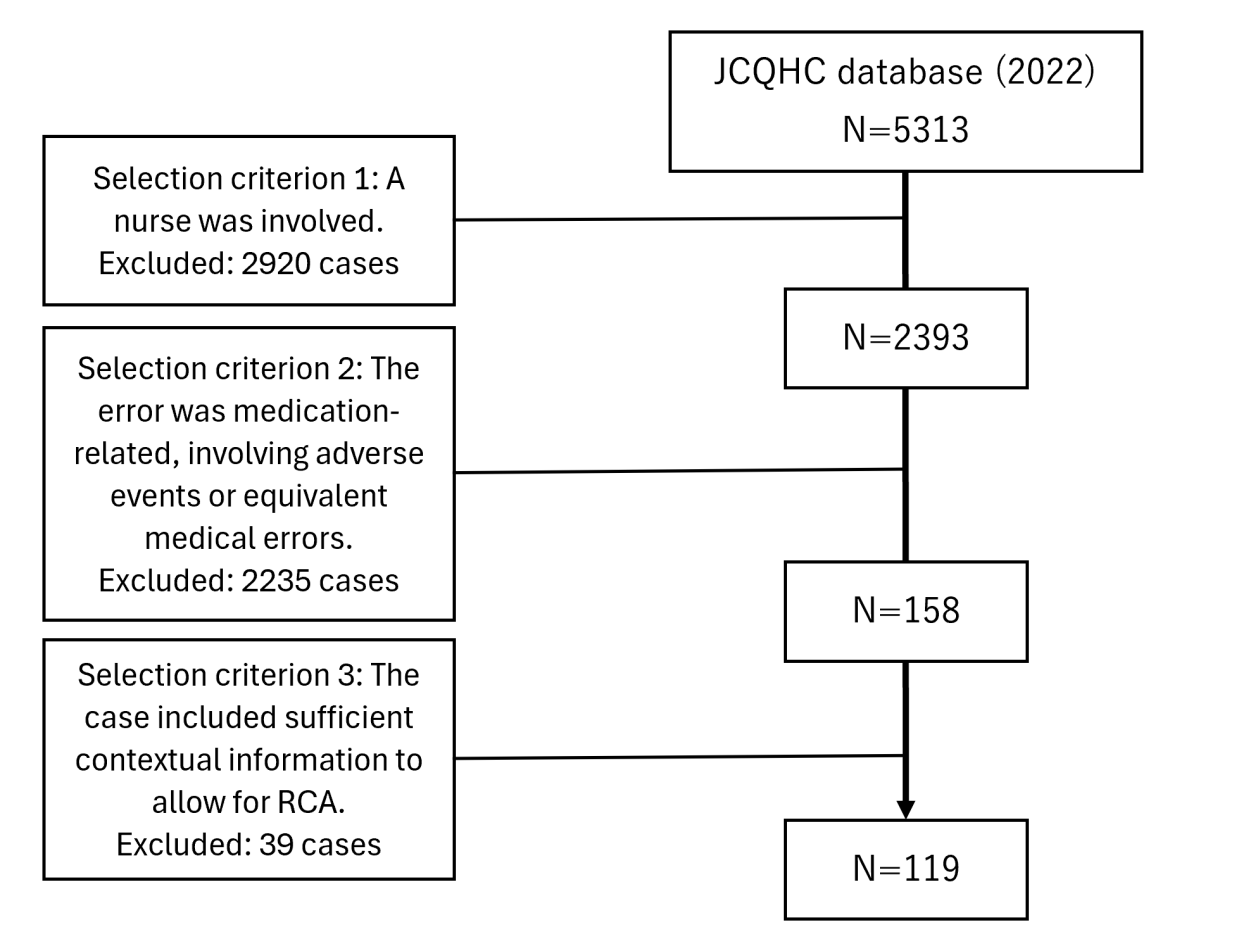
Data selection process. JCQHC: Japan Council for Quality Health Care; RCA: root cause analysis.

The distribution of the 119 incidents by clinical department was as follows. A total of 27 (22.7%) incidents occurred in surgical departments: neurosurgery (n=7 cases, 5.9%), orthopedic surgery (n=5 cases, 4.2%), gastrointestinal surgery (n=3 cases, 2.5%), cardiovascular surgery (n=1 case, 0.8%), plastic surgery (n=1 case, 0.8%), and oral and maxillofacial surgery (n=1 case, 0.8%). An additional 11 (9.2%) incidents were categorized as “other surgical departments,” referring to cases documented only as “surgery” or from mixed surgical wards (Figure 1).

A total of 47 (39.5%) incidents occurred in internal medicine departments: cardiology (n=7, 5.9%), hematology (n=6, 5.0%), gastroenterology (n=5, 4.2%), pulmonology (n=3, 2.5%), otorhinolaryngology (n=3, 2.5%), metabolism/endocrinology (n=3, 2.5%), neurology (n=2, 1.7%), dermatology (n=1, 0.8%), rheumatology (n=1, 0.8%), oncology (n=1, 0.8%), and general internal medicine (n=1, 0.8%). Another 14 (11.8%) incidents were categorized as “other internal medicine departments,” referring to cases recorded only as “internal medicine” or from mixed internal medicine wards.

In addition, 10 (8.4%) incidents occurred in the intensive care unit, 7 (5.9%) in the emergency room, and 4 (3.4%) in the neonatal intensive care unit. Other clinical areas included obstetrics and gynecology (11 incidents), pediatrics (9 incidents), psychiatry (1 incident), and palliative care (1 incident).

The distribution of incident locations was as follows. Patient rooms and wards accounted for the majority of cases (n=97 incidents, 81.5%), followed by outpatient clinics (n=12, 10.1%). Other locations were operating rooms (n=5, 4.2%), radiology examination rooms (n=3, 2.5%), dialysis units (n=1, 0.8%), cardiac catheterization laboratories (n=1, 0.8%), and patients’ homes (n=1, 0.8%).

Medication-related errors were classified into 10 event types, and 23 contributing factors were extracted (Table 1). Descriptive statistics by the unit experience group are summarized in Table 2. In the adaptation group (n=33), defined as nurses with less than 1 year of current unit experience, 8 (24.2%) had 5 to 9 years’ experience as registered nurses and 15 (45.5%) had ≥10 years. These findings indicated that this group was not composed solely of nurses with limited clinical experience.

**Table 2.** Summary statistics of nodes categorized by unit adaptation.

Node	All (N=119), n (%)	Low-adaptation model (n=33), n (%)	Moderate-adaptation model (n=36), n (%)	High-adaptation model (n=50), n (%)
Event^a^				
Wrong dose	43 (36.1)	15 (46)	10 (28)	18 (36)
Wrong patient	22 (18.5)	4 (12)	8 (22)	10 (20)
Improper mixing	15 (12.6)	5 (15)	6 (17)	4 (8)
Wrong or unnecessary administration	13 (10.9)	4 (12)	4 (11)	5 (10)
Overlooking contraindication	11 (9.2)	2 (6)	5 (14)	4 (8)
Accidental ingestion/overdose	7 (5.9)	1 (3)	3 (8)	3 (6)
Incorrect storage	6 (5.0)	1 (3)	1 (3)	4 (8)
Wrong timing	4 (3.4)	2 (6)	0	2 (4)
Default of medication discontinue	4 (3.4)	2 (6)	1 (3)	1 (2)
Wrong route	2 (1.7)	0	0	2 (4)
Attributional factors				
Years as registered nurse				
<5	39 (32.8)	10 (30)	18 (50)	11 (22)
5‐9	28 (23.5)	8 (24)	7 (19)	13 (26)
≥10	52 (43.7)	15 (46)	11 (31)	26 (52)
System factor				
Failure to detect by the electronic system	7 (5.9)	2 (6)	2 (6)	3 (6)
Conditional factors				
Weekday/holiday (during holiday)	24 (20.2)	6 (18)	9 (25)	9 (18)
Work time				
9 AM-4 PM	54 (45.4)	18 (55)	13 (36)	23 (46)
4 PM-9 PM	31 (26.1)	8 (24)	11 (31)	12 (24)
9 PM-9 AM	34 (28.6)	7 (21)	12 (33)	15 (30)
Day shift/night shift (during night shift)	65 (54.6)	15 (46)	23 (64)	27 (54)
Patients’ age (y)				
<20	12 (10.1)	4 (12)	2 (6)	6 (12)
20‐59	34 (28.6)	10 (30)	14 (39)	10 (20)
≥60	73 (61.3)	19 (58)	20 (56)	34 (68)
Time pressure	42 (35.3)	13 (39)	12 (33)	17 (34)
Sudden change in patients’ schedule	10 (8.4)	3 (9)	1 (3)	6 (12)
Failure of doctor’s orders	10 (8.4)	1 (3)	2 (6)	7 (14)
Patients who do not follow instructions	13 (10.9)	4 (12)	6 (17)	3 (6)
Knowledge and behavioral factors				
Failure to confirm the 5 rights	59 (49.6)	14 (42)	20 (56)	25 (50)
Assumptions and forgetfulness	47 (39.5)	12 (36)	14 (39)	21 (42)
Invalid double check	40 (33.6)	14 (42)	11 (31)	15 (30)
Improper use of instruments and equipment	35 (29.4)	6 (18)	13 (36)	16 (32)
Insufficient knowledge of medications	27 (22.7)	11 (33)	6 (17)	10 (20)
Unfamiliarity with operations of medications	23 (19.3)	10 (30)	7 (19)	6 (12)
Failure of information communication	23 (19.3)	7 (21)	10 (28)	6 (12)
Improper handling of medications	13 (10.9)	5 (15)	4 (11)	4 (8)
Failure of monitoring	13 (10.9)	3 (9)	5 (14)	5 (10)
Task interruption	11 (9.2)	3 (9)	0	8 (16)
Non-reconfirming of inappropriate doctor’s orders	10 (8.4)	2 (6)	2 (6)	6 (12)
Lack of explanation to patients	4 (3.4)	2 (6)	2 (6)	0
Miscalculation	4 (3.4)	0	1 (3)	3 (6)

a There were cases where multiple error events were segregated from a single incident report.

### Comparison of Models by Adaptation Levels

The BNM results for the 3 adaptation groups (low, moderate, and high) are presented in MultimediaAppendices 1^3^, and the comparison of their structural characteristics is summarized in Table 3. In each model, the incident event was positioned at the lowest level, while the most distal contributing factors were represented as level 1. The average entropy of the incident events ranged from 0.44 to 0.76 in the low-adaptation model, from 0.55 to 0.78 in the moderate-adaptation model, and from 0.37 to 0.75 in the high-adaptation model. In the low-adaptation model (Multimedia Appendix 1), the structure consisted of up to 5 layers. Four nodes were excluded from the model: 2 event nodes (“Incorrect storage” and “Wrong route”) and 2 factor nodes (“Patients who do not follow instructions” and “Miscalculation”). Three factor nodes were included in the model but were not connected to any event nodes: “Lack of explanation to patients,” “Failure of information communication,” and “Time pressure.”

**Table 3.** Comparison of model structures.

Comparison criteria	Low-adaptation model (n=33)	Moderate-adaptation model (n=36)	High-adaptation model (n=50)
Maximum number of layers	5	4	4
Number of events not included in the model	2	2	1
Node	Incorrect storage and wrong route	Wrong timing and wrong route	Wrong timing
Number of factors not included in the model	2	2	5
Node	Patients who do not follow instructions, miscalculation	Year as registered nurse, task interruption	Time pressure, assumptions and forgetfulness, failure of monitoring, lack of explanation to patients, miscalculation
Number of factors not related to events	3	7	5
Node	Time pressure, failure of information communication, and lack of explanation to patients	Failure to detect by the electronic system, assumptions and forgetfulness, invalid double check, failure of information communication, non-reconfirming of inappropriate doctor’s orders, lack of explanation to patients, miscalculation	Work time, failure of doctor’s orders, insufficient knowledge of medications, failure of information communication, non-reconfirming of inappropriate doctor’s orders

The moderate-adaptation model (Multimedia Appendix 2) had a maximum of 4 layers, with 4 nodes excluded from the model: 2 event nodes (“Wrong timing” and “Wrong route”) and 2 factor nodes (“Years as registered nurse” and “Task interruption”). Seven factor nodes were included in the model but were not connected to any event nodes: “Lack of explanation to patients,” “Miscalculation,” “Failure of information communication,” “Failure to detect by the electronic system,” “Invalid double check,” “Assumptions and forgetfulness,” and “Non-reconfirming of inappropriate doctor’s orders.”

In the high-adaptation model (Multimedia Appendix 3), the structure consisted of up to 4 layers. Six nodes were excluded from the model: one event node (“Wrong timing”) and 5 factor nodes (“Time pressure,” “Assumptions and forgetfulness,” “Miscalculation,” “Lack of explanation to patients,” and “Failure of monitoring”). Five factor nodes were included in the model but were not connected to any event nodes: “Insufficient knowledge of medications,” “Failure of information communication,” “Work time,” “Failure of doctor’s orders,” and “Non-reconfirming of inappropriate doctor’s orders.”

The node “Years as registered nurse” was included in both the low- and high-adaptation models (MultimediaAppendices 1  3). In the low-adaptation model, all categories except nurses with 5 to 9 years were positively associated with “Failure to confirm the five rights,” and nurses with ≥10 years were positively associated with “Wrong or unnecessary administration.” In the high-adaptation model, nurses with 5 to 9 years were positively associated with “Failure of information communication” and “Invalid double check,” whereas all categories except nurses with less than 5 years were positively associated with “Wrong dose.”

## Discussion

### Key Results

This hypothesis-generating study examined whether years of experience of nurses in their current unit, used as an indicator of unit adaptation, might be related to medication-related errors. While prior qualitative studies have highlighted the importance of adaptation [40], our mixed methods approach using an AI-based BNM suggests the potential for a more objective and quantitative assessment.

Incident causation inherently involves uncertainty because incidents arise from complex interactions among contextual and behavioral factors rather than predetermined outcomes. The moderate to slightly high entropy values observed in this study reflect this uncertainty and should be interpreted as relative indicators. Future studies with larger samples and additional variables, such as work environment factors, may help improve model stability and reliability.

BNM integrates expert judgment while reducing subjectivity through probabilistic modeling, differing from approaches that rely solely on RCA. Consistent with the Reason Swiss Cheese Model [41], our analysis visualized a multilayered structure of medication-related errors. Especially in the low-adaptation model, unsafe conditions and behaviors appeared to precede subsequent unsafe actions, suggesting a potential chain of events. The findings provide preliminary insights into how unit adaptation may influence error structures although this study is limited by its sample size and the interpretive constraints inherent to probabilistic modeling.

### Objectivity of RCA, Conceptual Validity, Causality, and Robustness of Hierarchical Structure Models

International studies on medication incidents have mainly reported that wrong dose errors account for a large proportion of events and that adherence to the “five rights” is a critical factor [37]. The descriptive statistics of incident events and contributing factors in the present study were generally consistent with these prior findings. Although the existing literature is limited, no notable bias was observed in the dataset used in this study.

With respect to RCA, factor extraction was deliberately restricted to elements that could be objectively derived from narrative descriptions; subjective elements prone to inconsistent reporting—such as psychological states and perceptions—were excluded. Furthermore, each incident event and its contributing factors were coded using predefined operational definitions to safeguard objectivity in the extraction process.

For the BNM, relationships among factors were specified with reference to human factors theory, drawing on frameworks [37,39]. These specifications informed the blacklist constraints, thereby supporting the conceptual validity of the resulting network structures.

Regarding the hierarchical structure models, the intent was not to establish definitive causal relationships but to conduct a hypothesis-generating exploration. All nurse knowledge and behavioral factors identified through RCA were treated as potential contributors, and the BNM was used to examine shared structural patterns and differences by unit experience. A greedy algorithm guided by the Akaike information criterion was selected to detect weak associations and latent risks without excluding relevant links, allowing modeling even with a relatively small sample size. However, the limited sample size constrains model stability and robustness, and the results should be interpreted as provisional hypotheses rather than confirmed evidence. The models do not determine causal relationships among factors or between factors and incidents. Future studies should increase the sample size and conduct cross-regional analyses to empirically test the hypotheses generated here.

### Effect of Adaptation on Patient Safety

As the concept of adaptive performance suggests, nurses’ adaptation to their unit is expected to enhance their problem-solving abilities, predictive practice skills, behavioral flexibility, and adjustment to the organizational culture and physical environment in highly uncertain clinical settings [19]. The findings of this study appear to be consistent with these expectations.

As shown in Table 2, incident-related factors did not decline sharply with increasing adaptation. However, the comparison of the 3 model structures (MultimediaAppendices 1^3^) suggests that the overall number of layers leading to incident occurrence becomes fewer as adaptation increases. One possible interpretation is that the low-adaptation group shares more homogeneous incident pathways, making certain unsafe conditions more likely to trigger errors. This trend underscores the importance of population-level patient safety education that supports early adaptation among novice and newcomer nurses.

Table 3 further shows that several factors—such as “Work time,” “Failure of information communication,” and “Non-reconfirming of inappropriate doctor’s orders”—were not directly connected to incidents in the high- and moderate-adaptation models. Other factors, including “Time pressure,” “Insufficient knowledge of medications,” and “Lack of explanation to patients,” were not included in the models at all. These patterns suggest two potential effects of unit adaptation: (1) improved adaptation to work processes, such as anticipatory multitasking, increased psychological resilience, acquisition of unit-specific pharmacological knowledge, and the ability to provide appropriate patient explanations [42]; and (2) enhanced adaptation to organizational culture, including smoother communication with colleagues and other health care professionals [43,44]. These interpretations remain tentative, and larger samples are needed to assess the stability and robustness of these structural differences.

Across all models, a consistent structure emerged involving “Wrong patient” and its associated factors—“Failure to confirm the 5 rights” and “Improper use of instruments and equipment.” Addressing these issues will likely require approaches beyond those focused solely on promoting unit adaptation.

### Considerations Regarding the Impact of Extensive Clinical Experience on Patient Safety

Previous studies on clinical experiences have primarily focused on patient safety measures for novice nurses with limited experience [22-25]. However, the inclusion of the node “Years as registered nurse” in both the low- and high-adaptation models indicated that extensive clinical experiences did not necessarily contribute to reducing errors. Instead, positive associations were observed with behaviors such as “Failure to confirm the five rights” and “Invalid double check,” suggesting lapses in verification behaviors.

One possible explanation is the emergence of inappropriate adaptation, in which safety behaviors become ritualized and lose their intended effectiveness. Another possibility is the presence of a cognitive bias whereby experienced nurses are assumed by other staff to be less likely to make errors, leading to the invalidation of double-checking procedures [45]. Although the potential negative effects of extensive clinical experiences have rarely been considered, and the present findings do not allow us to conclude a causal relationship between greater years of experience and errors in verification behaviors, the consistent adherence to safety-verification practices should apply to all nurses, regardless of their level of experience.

### Practical Implications

The most significant contribution of this study lies in suggesting that, while previous research has emphasized the lack of clinical experiences among novice nurses as a major issue for patient safety [22-25], our analysis of real-world data indicates that challenges in adapting to clinical units may also underlie this issue. This represents a novel application of AI in nursing education [46]. These findings underscore the potential importance of supporting nurses’ adaptation to their assigned units—not only for newly graduated nurses but also for those who have recently transferred to new settings. Educational approaches that emphasize adaptation may also play a meaningful role in the training of other health care professionals. Further hypothesis-testing research will be required.

As possible practical interventions, intensive orientation programs focusing on unit-specific workflows during the initial assignment phase, along with educational initiatives aimed at improving medication knowledge and fostering safe practices, may be effective. In the field of safety behavior training, virtual reality programs have been developed and applied to surgical education [47]. Following this example, scenario-based simulation training can be designed using the results of the present models to reproduce situations in which unsafe nursing behaviors are likely to cascade.

Furthermore, the models presented in this study can be used as feedback tools for safety management and education, reflecting individual differences in clinical experiences and adaptation levels. Although adverse events and medical errors are often influenced by multiple latent factors [37], postevent instructions tend to focus primarily on direct causes due to hindsight bias. The multilayered structures revealed in the current models may help overcome this bias by encouraging more comprehensive and proactive safety-management strategies.

### Limitations and Future Work

We conducted RCA based on narrative descriptions of reported medical errors. Therefore, situations or latent factors unrecognized by reporting nurses may not have been captured in the analysis. In particular, nurses with extensive clinical experiences often care for critically ill patients or manage heavier workloads, which may serve as underlying factors contributing to counterproductive behaviors, such as task shortcuts or neglect of verification behaviors [48]. However, this limitation is not unique to this study but rather reflects a structural issue common to existing incident reporting systems worldwide. Indeed, prior studies on nurse fatigue risk management have rarely incorporated variables such as patient load and workload in a systematic manner. In addition, even in routine clinical practice, systems for consistently recording indicators such as the number of patients per nurse or patient acuity for the purpose of workload analysis are often lacking. If quantitative approaches to analyzing incident reports—such as those explored in our study—become more established, they may contribute to future international discussions on revising reporting standards.

Another limitation concerns the balance between global and local optimization in the analysis; that is, the extent to which institutional characteristics are incorporated depends on the research purpose and may affect the generalizability of the results [2]. In this study, organizational factors specific to individual institutions could not be extracted from the report narratives. Future research should compare data across institutions and design unit-specific or institution-specific training programs, followed by an evaluation of their effectiveness and acceptability [49].

The findings from the hierarchical network models in this study should be regarded as exploratory and hypothesis-generating rather than definitive. Future work is needed to expand the sample and to evaluate the stability and robustness of the model structures through random-sampling approaches, such as analyzing pre- and post-COVID-19 datasets [50] and employing bootstrap methods [51].

The variable “Years of nursing experience” spans a broad continuous range, so it was converted into categorical data using 5-year intervals, following classifications commonly used in clinical ladder systems [21,33]. This categorization, however, is not definitive. Greater experiences may also reflect age-related factors, and although declines in work ability with age are well recognized in occupational health [52], they have rarely been examined in medical or nursing contexts. Further research is needed to clarify how nursing experience relates to work performance and to determine more appropriate ways of grouping experience levels.

### Conclusions

This hypothesis-generating study applied an AI-based BNM to examine structural patterns underlying medication-related errors, demonstrating the value of a quantitative, probabilistic approach that goes beyond the traditional RCA. The use of BNM represents a novel methodological contribution in patient safety research by combining expert judgment with reduced subjectivity and enabling structural comparison across experience groups. The findings suggest that unit adaptation plays an important role in shaping error pathways: low-adaptation nurses showed more layered and homogeneous structures leading to unsafe actions, indicating a higher vulnerability to incident chains. In contrast, total years of nursing experience did not uniformly reduce risk and may even relate to lapses in verification behaviors. Together, these results highlight the need to support adaptation not only among new graduates but also among nurses transitioning to new units, while reinforcing verification practices across all experience levels. Further empirical studies are needed to validate these preliminary hypotheses.

## Supplementary material

10.2196/87436 Multimedia Appendix 1 The low-adaptation model.

10.2196/87436 Multimedia Appendix 2 The moderate-adaptation model.

10.2196/87436 Multimedia Appendix 3 The high-adaptation model.

## Abbreviations

AI: artificial intelligence
BNM: Bayesian network modeling
JCQHC: Japan Council for Quality Health Care
RCA: root cause analysis
WHO: World Health Organization

## References

[1] Hervatis V, Loe A, Barman L, O’Donoghue J, Zary N (2015). A conceptual analytics model for an outcome-driven quality management framework as part of professional healthcare education. JMIR Med Educ.

[2] Mohamed B, Fahy BG (2022). The impact of data-driven learning on graduate medical education. J Clin Anesth.

[3] Lin L, Zhou D, Wang J, Wang Y (2024). A systematic review of big data driven education evaluation. Sage Open.

[4] Patient safety. World Health Organization.

[5] Tella S, Liukka M, Jamookeeah D, Smith NJ, Partanen P, Turunen H (2014). What do nursing students learn about patient safety? An integrative literature review. J Nurs Educ.

[6] Lee SE, Morse BL, Kim NW (2022). Patient safety educational interventions: a systematic review with recommendations for nurse educators. Nurs Open.

[7] (2021). The essentials: core competencies for professional nursing education. https://www.aacnnursing.org/Portals/0/PDFs/Publications/Essentials-2026.pdf.

[8] Wittmann-Price RA, Wittmann RA, Gittings KK (2020). Fast Facts About Competency-Based Education in Nursing How to Teach Competency Mastery.

[9] Grigsby K, Billings DM, Halstead JA (2019). Teaching in Nursing: A Guide for Faculty.

[10] Currie L, Watterson L (2007). Challenges in delivering safe patient care: a commentary on a quality improvement initiative. J Nurs Manag.

[11] Henneman EA, Roche JP, Fisher DL (2010). Error identification and recovery by student nurses using human patient simulation: opportunity to improve patient safety. Appl Nurs Res.

[12] Rinaldi K, Messer M, Hanson A, Chan J (2025). Utilization of backward design in health professional education: a rapid review. J Prof Nurs.

[13] Hodkinson A, Tyler N, Ashcroft DM (2020). Preventable medication harm across health care settings: a systematic review and meta-analysis. BMC Med.

[14] Panagioti M, Khan K, Keers RN (2019). Prevalence, severity, and nature of preventable patient harm across medical care settings: systematic review and meta-analysis. BMJ.

[15] Sugimura N, Ogasawara K (2024). Events related to medication errors and related factors involving nurses’ behavior to reduce medication errors in Japan: a Bayesian network modeling-based factor analysis and scenario analysis. J Educ Eval Health Prof.

[16] Griffin MA, Neal A, Parker SK (2007). A new model of work role performance: positive behavior in uncertain and interdependent contexts. Acad Manage J.

[17] Sugimura N, Sato M, Sumi N, Yano R (2023). Validity and reliability of the Japanese version of the Nursing Performance Instrument. Jpn J Nurs Sci.

[18] Dawson D, Chapman J, Thomas MJW (2012). Fatigue-proofing: a new approach to reducing fatigue-related risk using the principles of error management. Sleep Med Rev.

[19] Gander P, Hartley L, Powell D (2011). Fatigue risk management: organizational factors at the regulatory and industry/company level. Accid Anal Prev.

[20] Benner P, Tanner C, Chesla C (1996). Expertise in Nursing Practice: Caring, Clinical Judgment, and Ethics.

[21] Slagle A, Wakim N, Gray SE (2023). A global examination of clinical ladder programs—a synthesis of commonalities and opportunities for standardization. Worldviews Evid Based Nurs.

[22] Hou M, Wei W, Wang S, Hu J, Chen Y (2025). Challenge-hindrance stressors and novice nurses’ safety behaviour: the mediating role of regulatory focus and the moderating role of workplace spirituality. J Adv Nurs.

[23] Labrague LJ, De Los Santos JAA (2020). Transition shock and newly graduated nurses’ job outcomes and select patient outcomes: a cross-sectional study. J Nurs Manag.

[24] Yan J, Li L, Li J (2022). Stepwise interactive situated training program for young nurses’ safety behavior and interrupted coping behavior. Healthcare (Basel).

[25] Ebright PR, Urden L, Patterson E, Chalko B (2004). Themes surrounding novice nurse near-miss and adverse-event situations. J Nurs Adm.

[26] Battles JB, Shea CE (2001). A system of analyzing medical errors to improve GME curricula and programs. Acad Med.

[27] Outline [Article in Japanese]. Japan Council for Quality Health Care.

[28] Fukada M (2018). Nursing competency: definition, structure and development. Yonago Acta Med.

[29] Mrayyan MT, Abunab HY, Abu Khait A (2023). Competency in nursing practice: a concept analysis. BMJ Open.

[30] Almarwani AM, Alzahrani NS (2023). Factors affecting the development of clinical nurses’ competency: a systematic review. Nurse Educ Pract.

[31] Lin YW, Ni CF, Hsu SF, Tsay SL, Tung HH (2024). Effects of length of employment and head nurse leadership style on the clinical competency of staff nurses in Taiwan. J Nurs Res.

[32] Saiga M, Yamamoto Y, Okuda R, Fukada M (2024). Relationship between clinical nursing competence and work environment by career stage for nurses with 1-10 years of clinical experience. Yonago Acta Med.

[33] Career philosophy [Article in Japanese]. Hokkaido University Hospital Nursing Department.

[34] Campbell JP, Dunnette MD, Hough LM (1990). Handbook of Industrial and Organizational Psychology.

[35] Viswesvaran C, Ones DS (2000). Perspectives on models of job performance. Int J Sel Assess.

[36] Koopmans L, Bernaards CM, Hildebrandt VH, Schaufeli WB, de Vet Henrica CW, van der Beek AJ (2011). Conceptual frameworks of individual work performance: a systematic review. J Occup Environ Med.

[37] Mitchell RJ, Williamson A, Molesworth B (2015). Use of a human factors classification framework to identify causal factors for medication and medical device-related adverse clinical incidents. Saf Sci.

[38] Zhao L, Wang X, Qian Y (2012). Analysis of factors that influence hazardous material transportation accidents based on Bayesian networks: a case study in China. Saf Sci.

[39] Lee JY, Huang CH, Sie YA, Yang PC, Su CC, Chang JT (2025). Applying the human factors analysis and classification system within root cause analysis to prevent medical errors and enhancing patient safety culture: insights from a medical center. Int J Qual Health Care.

[40] Jung S, Park J (2025). Educational needs for medication safety competence among nurses by clinical ladder stage. PLoS One.

[41] Wiegmann DA, Wood LJ, Cohen TN, Shappell SA (2022). Understanding the “Swiss cheese model” and its application to patient safety. J Patient Saf.

[42] Alsabri M, Boudi Z, Lauque D (2022). Impact of teamwork and communication training interventions on safety culture and patient safety in emergency departments: a systematic review. J Patient Saf.

[43] Keshtkar L, Bennett-Weston A, Khan AS (2025). Impacts of communication type and quality on patient safety incidents: a systematic review. Ann Intern Med.

[44] Kwame A, Petrucka PM (2021). A literature-based study of patient-centered care and communication in nurse-patient interactions: barriers, facilitators, and the way forward. BMC Nurs.

[45] Eldor L, Hodor M, Cappelli P (2023). The limits of psychological safety: nonlinear relationships with performance. Organ Behav Hum Decis Process.

[46] Buchanan C, Howitt ML, Wilson R, Booth RG, Risling T, Bamford M (2021). Predicted influences of artificial intelligence on nursing education: scoping review. JMIR Nurs.

[47] Mazur L, Butler L, Mitchell C (2025). Effect of immersive virtual reality teamwork training on safety behaviors during surgical cases: nonrandomized intervention versus controlled pilot study. JMIR Med Educ.

[48] Rotundo M, Sackett PR (2002). The relative importance of task, citizenship, and counterproductive performance to global ratings of job performance: a policy-capturing approach. J Appl Psychol.

[49] Lee JWY, Tan JY, Bello F (2025). Technology acceptance model in medical education: systematic review. JMIR Med Educ.

[50] Clari M, Luciani M, Conti A (2021). The impact of the COVID-19 pandemic on nursing care: a cross-sectional survey-based study. J Pers Med.

[51] Broom BM, Do KA, Subramanian D (2012). Model averaging strategies for structure learning in Bayesian networks with limited data. BMC Bioinformatics.

[52] von Bonsdorff ME, Kokko K, Seitsamo J (2011). Work strain in midlife and 28-year work ability trajectories. Scand J Work Environ Health.

