# Supplementary figures and images for: Medication-Related Errors Among Nurses by Unit Adaptation Levels: Bayesian Network–Based Exploratory Study
Source: JMIR Nurs. 2026 Jul 7;9:e87436. doi: 10.2196/87436 (PMC13340574; doi:10.2196/87436)

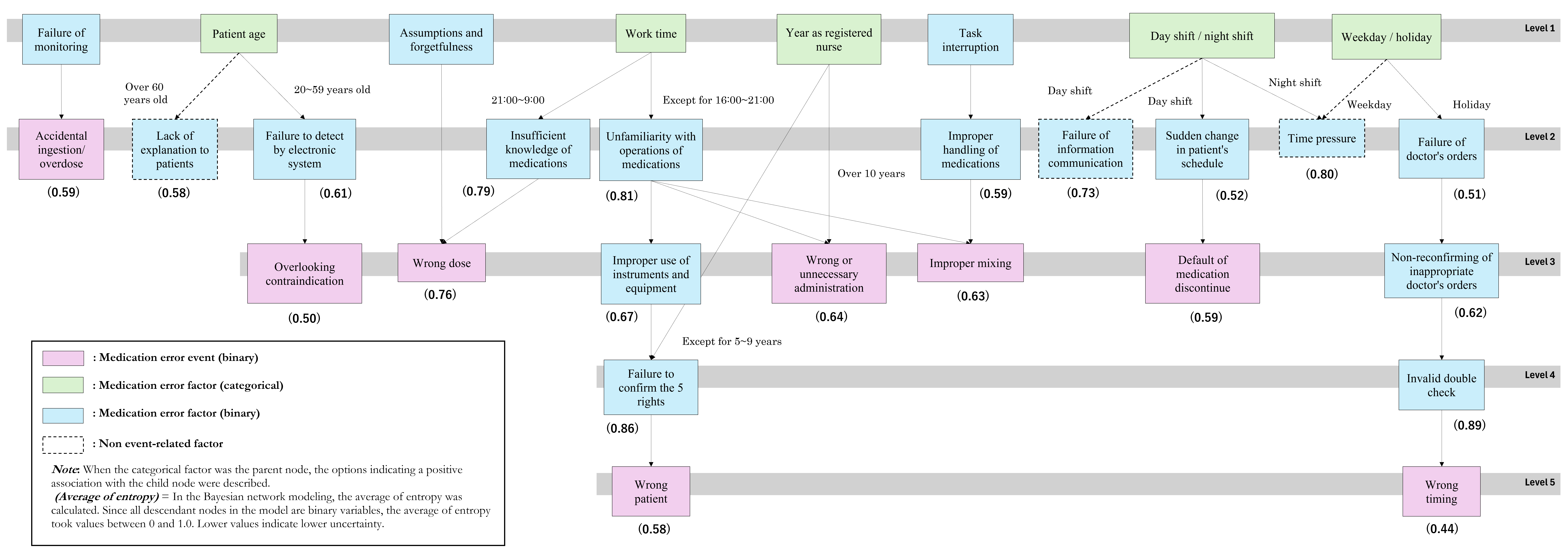

Supplement: Multimedia Appendix 1 [file nursing-v9-e87436-s001.png]

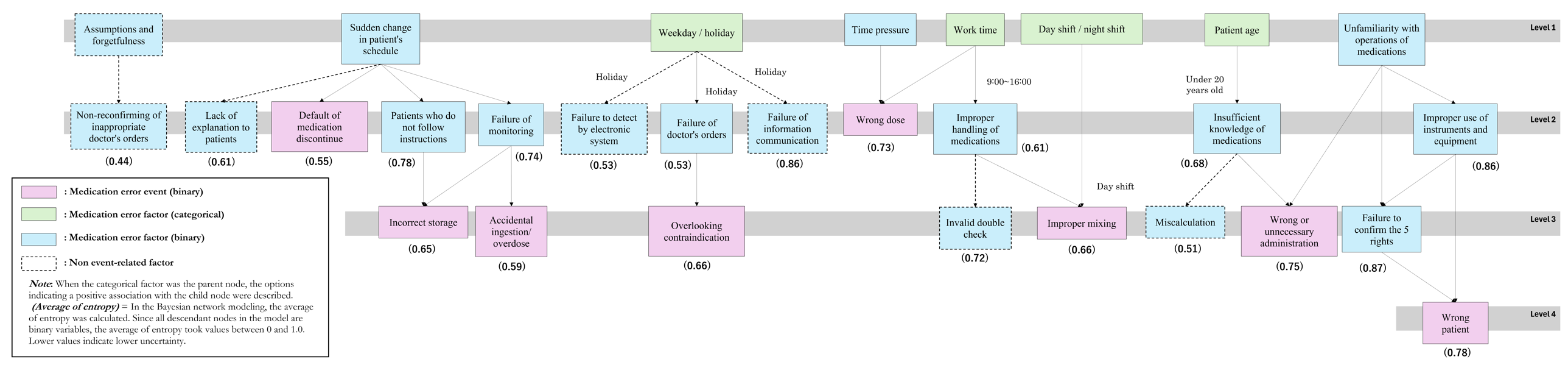

Supplement: Multimedia Appendix 2 [file nursing-v9-e87436-s002.png]

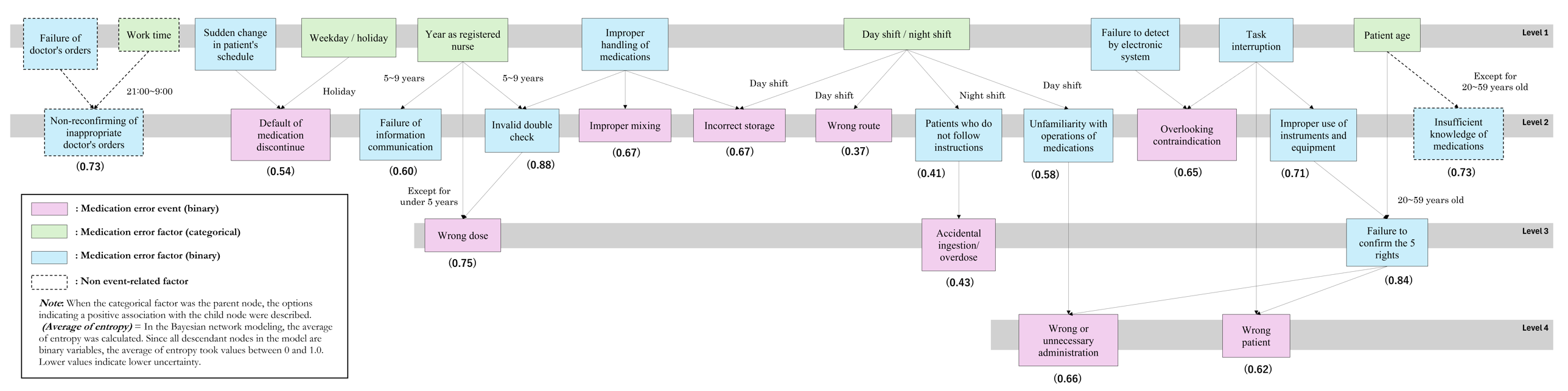

Supplement: Multimedia Appendix 3 [file nursing-v9-e87436-s003.png]
